# Wheat height monitoring from GPS/BDS reflected signals using pseudorange and dual-frequency carrier phase observables

**DOI:** 10.3389/fpls.2025.1717765

**Published:** 2025-12-17

**Authors:** Mingming Sui, Zhongpei Guan, Peng Cui

**Affiliations:** 1 College of Civil Engineering, Nanjing Forestry University, Nanjing, China; 2 Jiangsu Yitu Geographic Information Technology Co., Ltd., Yangzhou, China; 3 Department of Applied Physics and Electronics, Umeå Universitet, Umeå, Sweden

**Keywords:** ground-based GNSS-R, wheat height monitoring, pseudorange, carrier phase, multi-system and multi-satellite fusion

## Abstract

**Introduction:**

Ground-based Global Navigation Satellite System Reflectometry (GNSS-R) has recently emerged as a low-cost, continuous, and high-resolution technique for monitoring crop growth. However, conventional GNSS-R approaches that rely on signal-to-noise ratio (SNR) observables are limited by data availability, particularly from legacy receivers, and the effectiveness of observable combination methods in this context has not yet been established.

**Methods:**

This study presents the first successful attempt to retrieve wheat height using ground-based GNSS-R with code pseudorange and dual-frequency carrier phase observables. Six observable combination schemes from GPS and BDS were evaluated through a field experiment at the Fengqiu Agro-ecology Experimental Station in China. A GDD-parameterized Logistic growth model was employed as a continuous reference. A multi-system, multi-satellite fusion strategy was developed, incorporating principal frequency power weighting within each system and residual reciprocal weighting across systems.

**Results:**

The observable combination method effectively captured wheat growth dynamics. The best-performing combinations—GPS C5I+L5I+L2P and BDS C2I+L2I+L6I—achieved correlation coefficients (R) of 0.935 and 0.957, and RMSE values of 0.081 m and 0.086 m, respectively. Dual-system fusion further enhanced retrieval accuracy, reducing RMSE by 22.6% compared with the best single-system combination and by 34.6% relative to an SNR-based method.

**Discussion:**

These findings demonstrate the feasibility and superiority of pseudorange and dual-frequency carrier phase combinations for SNR-independent GNSS-R crop monitoring. The proposed strategy offers a robust, scalable, and accessible tool for precision agriculture and continuous crop growth tracking, particularly in contexts where SNR data are unavailable or unreliable.

## 1 Introduction

Wheat is a staple cereal crop of global importance, with its annual yield serving as a vital metric for agricultural productivity and food security planning. Plant height, a key phenotypic trait, offers significant value in this regard due to its strong correlation with biomass, lodging resistance, and final grain yield, establishing it as an essential variable for growth monitoring and yield prediction models (Franch et al., 2019; Hassan et al., 2019; Nduku et al., 2023). The increasing frequency and intensity of extreme weather events attributed to climate change, however, pose substantial threats to wheat production. Meteorological stressors like heavy rain and strong winds can induce lodging, which severely compromises crop development and reduces yields. Since anomalies in canopy height represent one of the earliest observable indicators of such stress, monitoring wheat height dynamics with high temporal resolution throughout the growing season becomes imperative. This capability is crucial for enhancing yield forecasting accuracy and developing effective early warning systems against crop failure.

For decades, the measurement of wheat height has primarily depended on manual techniques using simple tools such as rulers or meter sticks. Although straightforward, these methods are laborious, time-consuming, and fundamentally incapable of supporting continuous or high-frequency monitoring across large agricultural fields. In light of these constraints, recent studies have turned to advanced remote sensing technologies including Synthetic Aperture Radar (SAR) (Tiwari et al., 2020; Wang et al., 2024) and Light Detection and Ranging (LiDAR) (Jimenez-Berni et al., 2018) for estimating wheat height. Despite these technological advances, such approaches remain limited by high operational costs or insufficient temporal resolution, rendering them less feasible for real-time, large-scale monitoring of wheat growth (Liao et al., 2018).

In the past few years, the reflected signals from Global Navigation Satellite System (GNSS) are found to be a signal of opportunity to observe the earth surface, and this technology is called the Global Navigation Satellite System-Reflectometry (GNSS-R). It enables the estimation of relevant parameters by analyzing the variations in characteristics observed within the reflection signals (Rodriguez-Alvarez et al., 2023; Wang et al., 2025). According to the platform of receiving equipment, GNSS-R can be divided into three primary observation modes: spaceborne (Bu et al., 2024), airborne (Wu et al., 2025) and ground-based (Zheng et al., 2021). Spaceborne and airborne GNSS-R typically employ specially designed receivers to retrieve environmental parameters. While these platforms offer greater flexibility for monitoring specific regions of interest, they are often constrained by high costs and an inability to provide continuous observations. In contrast, ground-based GNSS-R can utilize data from geodetic quality receivers of existing GNSS networks, offering distinct advantages such as low incremental cost, long-term operation, and continuous data acquisition, making it particularly suitable for agricultural monitoring in fixed locations (Yang et al., 2024).

In ground-based GNSS-R, the signal-to-noise ratio (SNR) would emerge oscillations due to the interference signals. Then, the vertical distance from the receiver antenna to reflective medium surface can be measured from SNR oscillations, which is defined as reflector height (RH) and thus a series of applications have been derived such as snow depth retrieval, sea level retrieval and vegetation growth detection (Yu et al., 2022). As an important branch of land applications, wheat height retrieval has been widely investigated. A foundational study by Zhang et al. (2017) first proposed a vegetation-height retrieval algorithm using the dominant period of GPS L1 SNR observables, noting its sensitivity to height changes of at least one signal wavelength. Further building on this, another study by Zhang et al. (2021) comprehensively evaluated the potential of both BDS and GPS for retrieving soil moisture and characterizing vegetation growth, comparing Empirical Mode Decomposition (EMD) and wavelet algorithms. More recently, Sui et al. (2022) comprehensively evaluated the potential of multi-frequency SNR signals from GPS, BDS, GLONASS, and Galileo in monitoring wheat growth. By integrating multi-GNSS satellite signals, the accuracy of wheat height retrieval was significantly improved.

Building on prior advances in GNSS-R, wheat height retrieval from SNR oscillations is now relatively mature. However, SNR observables are not consistently available in raw GNSS data files, particularly for legacy receivers, which limits the operational applicability of SNR-based methods in long-term or historical networks (Zhang et al., 2020). In this context, maintaining the ability to monitor wheat growth without relying on SNR is both practically valuable and scientifically important. Fortunately, the linear combinations of GNSS observables (pseudorange and carrier phase) have been demonstrated effective in restore multipath and thus used to obtain RH. Ozeki and Heki (2012) proposed a dual-frequency phase combination with GPS L1/L2 signals, which is free of geometric terms, to estimate snow depth. However, the L4 linear combination of observables contains inter-frequency ionospheric terms, which deteriorate the snow depth retrieval performance. To directly eliminate the effects of ionospheric delays, Yu et al. (2015) adopted a triple-frequency phase combination. In addition, the combination of pseudorange and dual-frequency/single-frequency carrier phases also have been investigated (Yu et al., 2018; He et al., 2025).

However, the above-mentioned approaches have been applied primarily to sea level altimetry and snow depth estimation, with no systematic investigations reported for wheat height retrieval. The wheat field acts as a volume scatter, where signals are subject to attenuation, diffraction, and multiple interactions within the canopy layer, and this discrepancy could degrade the quality of the multipath observation and thus affect retrieval accuracy. Therefore, validating the feasibility and robustness of the combination method specifically for the complex scenario of crop monitoring is both necessary and valuable. In this study, the differences of individual system and frequency signals on wheat height retrieval are deeply analyzed, and multi-system and multi-satellite retrievals are fused to improve the performance. In addition, the retrievals are compared with simulations and *in-situ* records and for systematic evaluation, resulting in an efficient method for monitoring or providing early warning of wheat height variations.

## 2 Methodology

### 2.1 Observation geometry

**Figure 1:**
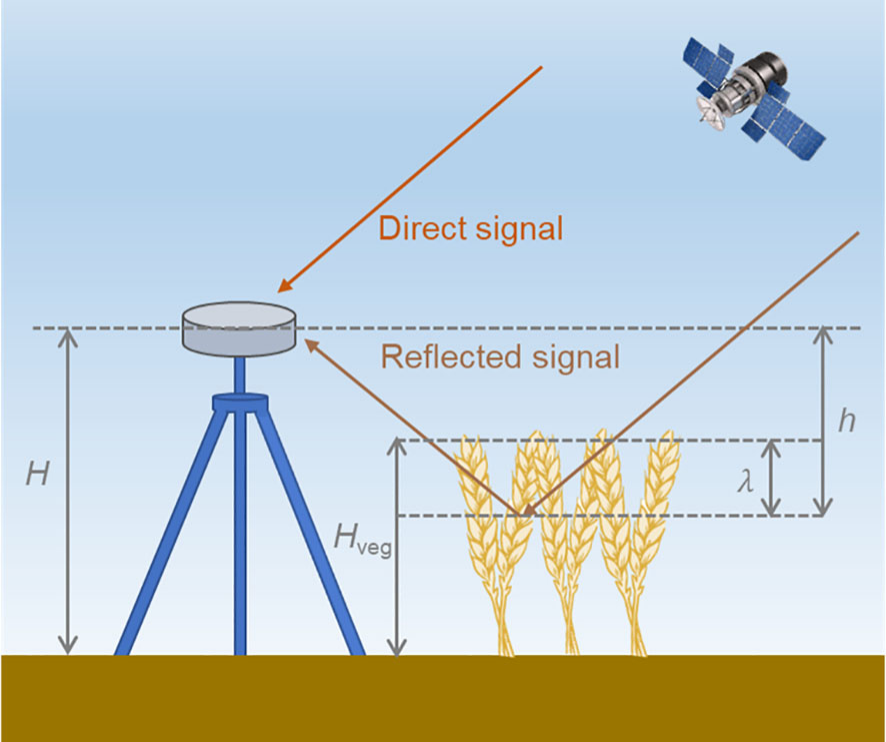
Schematic diagram of wheat height monitoring based on ground-based GNSS-R.

The principle of monitoring wheat height using based on ground-based GNSS-R technology is illustrated in **Figure 1**. Here,. represents the distance from the antenna phase center to the bare soil surface, while $H_{\text{veg}}$ represents the distance from the top of the wheat canopy to the soil. Additionally, h denotes the distance from the antenna phase center to the wheat canopy layer, factoring in signal penetration, which is measured directly by GNSS-R geometry.

Given the specific signal penetration capacity of GNSS signals through sparse wheat canopy, the height measured by GNSS-R ( h) typically encompasses the signal’s penetration depth within the vegetation layer. This necessitates calibration of the retrieval outcomes. Previous research suggests that the combination method cannot distinguish height variations within a signal wavelength. By incorporating the disparity between the antenna height above ground ( H) and the wheat height ( $H_{\text{veg}}$), along with an additional wavelength, a closer alignment with the measured distance ( h) can be achieved (Zhang et al., 2021). Consequently, the geometric model for wheat height retrieval via ground-based GNSS-R can be articulated as Equation 1:

(1) $$\begin{array}{c} H_{\text{veg}}=H-h+\lambda \end{array}$$

where λ denotes the wavelength of signal.

### 2.2 Observable combination method

The foundation of ground-based GNSS-R altimetry lies in multipath error. The approach adopted in this investigation involves a linear combination of code pseudorange and dual-frequency carrier phase observables to compute multipath observation. Taking GPS L1 and L2 signals as an illustration, the detailed calculation methodology is detailed below.

The pseudorange $\tilde{\rho }\left(t\right)\;\;$ and the carrier phase $\lambda \tilde{\varphi }\left(t\right)$ received by the receiver at epoch t can be expressed respectively as Equation 3 (Ozeki and Heki, 2012; Zhang et al., 2020):

(2) $$\begin{array}{c} \{\begin{array}{c} {\tilde{\rho }}_{1}\left(t\right)=\rho +I_{1}+T+\ell_{1}\left(t\right)\\ \lambda_{1}{\tilde{\varphi }}_{1}\left(t\right)=\rho -I_{1}+T+\beta_{1}\left(t\right)-\lambda_{1}N_{1}\\ \lambda_{2}{\tilde{\varphi }}_{2}\left(t\right)=\rho -I_{2}+T+\beta_{2}\left(t\right)-\lambda_{2}N_{2} \end{array} \end{array}$$

The subscripts in the equation denote the parameters for the L1 and L2 frequency bands, respectively, ρ represents the geometric range between the satellite and the receiver, ι  denotes the ionospheric delay, T is the tropospheric delay, λ is the carrier wavelength, ν  is the integer ambiguity, and ℓ and β​ represent the multipath errors in pseudorange and carrier phase observations, respectively. The approximate expressions for these errors are detailed as follows (Equations 3, 4):

(3) $$\begin{array}{c} l=\frac{\Delta \left(t\right)\alpha \cos(\delta \varphi \left(t\right))}{1+\alpha \cos(\delta \varphi \left(t\right))} \end{array}$$

(4) $$\begin{array}{c} \beta =\arctan\left(\frac{\alpha \alpha \sin(\delta \varphi \left(t\right))}{1+\alpha \cos(\delta \varphi \left(t\right))}\right) \end{array}$$

where α is the amplitude attenuation factor; Δ(t) and δφ(t) represent the path delay and phase delay of the reflected signal relative to the direct signal, respectively. Their expressions are given by Equations 5, 6:

(5) $$\begin{array}{c} \Delta \left(t\right)=2h\sin\theta \left(t\right) \end{array}$$

(6) $$\begin{array}{c} \delta \varphi \left(t\right)=\frac{4\pi h\sin\left(\theta \right)}{\lambda } \end{array}$$

where θ is the satellite elevation angle, and h is the distance from the antenna phase center to the wheat canopy layer. By jointly processing the fractional terms in the simultaneous equation set of Equation 2, the following expression is obtained, Equation 7:

(7) $$\begin{array}{c} \{\begin{array}{c} \begin{array}{c} {\tilde{\rho }}_{1}\left(t\right)-\lambda_{1}{\tilde{\varphi }}_{1}\left(t\right)=2I_{1}+\ell_{1}\left(t\right)-\beta_{1}\left(t\right)+\lambda_{1}N_{1}\\ {\tilde{\rho }}_{1}\left(t\right)-\lambda_{2}{\tilde{\varphi }}_{2}\left(t\right)=I_{1}+I_{2}+\ell_{1}\left(t\right)-\beta_{2}\left(t\right)+\lambda_{2}N_{2} \end{array} \end{array} \end{array}$$

After multiplying the numerator and denominator of Equation 7 by $\kappa_{1}=\frac{\lambda_{1}^{2}+\lambda_{2}^{2}}{\lambda_{1}^{2}-\lambda_{2}^{2}}$ and $\kappa_{2}=\frac{-2\lambda_{1}^{2}}{\lambda_{1}^{2}-\lambda_{2}^{2}}$ respectively and then adding and rearranging the terms, the following expression, Equation 8 is obtained:

(8) $$\begin{array}{c} {\tilde{\rho }}_{1}\left(t\right)+\kappa_{1}\lambda_{1}{\tilde{\varphi }}_{1}\left(t\right)+\kappa_{2}\lambda_{2}{\tilde{\varphi }}_{2}\left(t\right)=\\ -\left[\left(2\kappa_{1}+\kappa_{2}\right)I_{1}+\kappa_{2}I_{2}\right]+\left[\ell_{1}\left(t\right)+\kappa_{1}\beta_{1}\left(t\right)+\kappa_{2}\beta_{2}\left(t\right)\right]+C \end{array}$$

where $\;C=-\left(\kappa_{1}\lambda_{1}\nu_{1}+\kappa_{2}\lambda_{2}\nu_{2}\right)\;$ represents the combined integer ambiguity value from different frequency bands. Assumed that the cycle slips are compensated, the term C can be regarded as a constant and does not affect the frequency of the multipath-induced oscillations (Equation 8). Then, C can be removed by a detrending process (wavelet decomposition in this manuscript). According to the ionospheric formula, the ionospheric range delay is inversely proportional to the square of the frequency. Since frequency is inversely proportional to the wavelength of electromagnetic waves, it can be deduced that the ionospheric delay is proportional to the square of the wavelength, namely:

(9) $$\begin{array}{c} \;\;\frac{\iota_{1}}{\iota_{2}}=\frac{\lambda_{1}^{2}}{\lambda_{2}^{2}} \end{array}$$

Substituting Equation 9 into Equation 8 yields Equation 10:

(10) $$\begin{array}{c} \mu_{1,2}\left(t\right)=\;{\tilde{\rho }}_{1}\left(t\right)+\kappa_{1}\lambda_{1}{\tilde{\varphi }}_{1}\left(t\right)+\kappa_{2}\lambda_{2}{\tilde{\varphi }}_{2}\left(t\right)=\ell_{1}\left(t\right)+\kappa_{1}\beta_{1}\left(t\right)+\kappa_{2}\beta_{2}\left(t\right) \end{array}$$

According to the above equation, the multipath combination error of pseudorange and carrier phase $\mu_{1,2}$ can be obtained through a linear combination of pseudorange observations and dual-frequency carrier phase observations. h can be estimated from the oscillation frequency f of the combination series, as detailed in Equation 11:

(11) $$\begin{array}{c} f=2h/\lambda \end{array}$$

**Figure 2:**
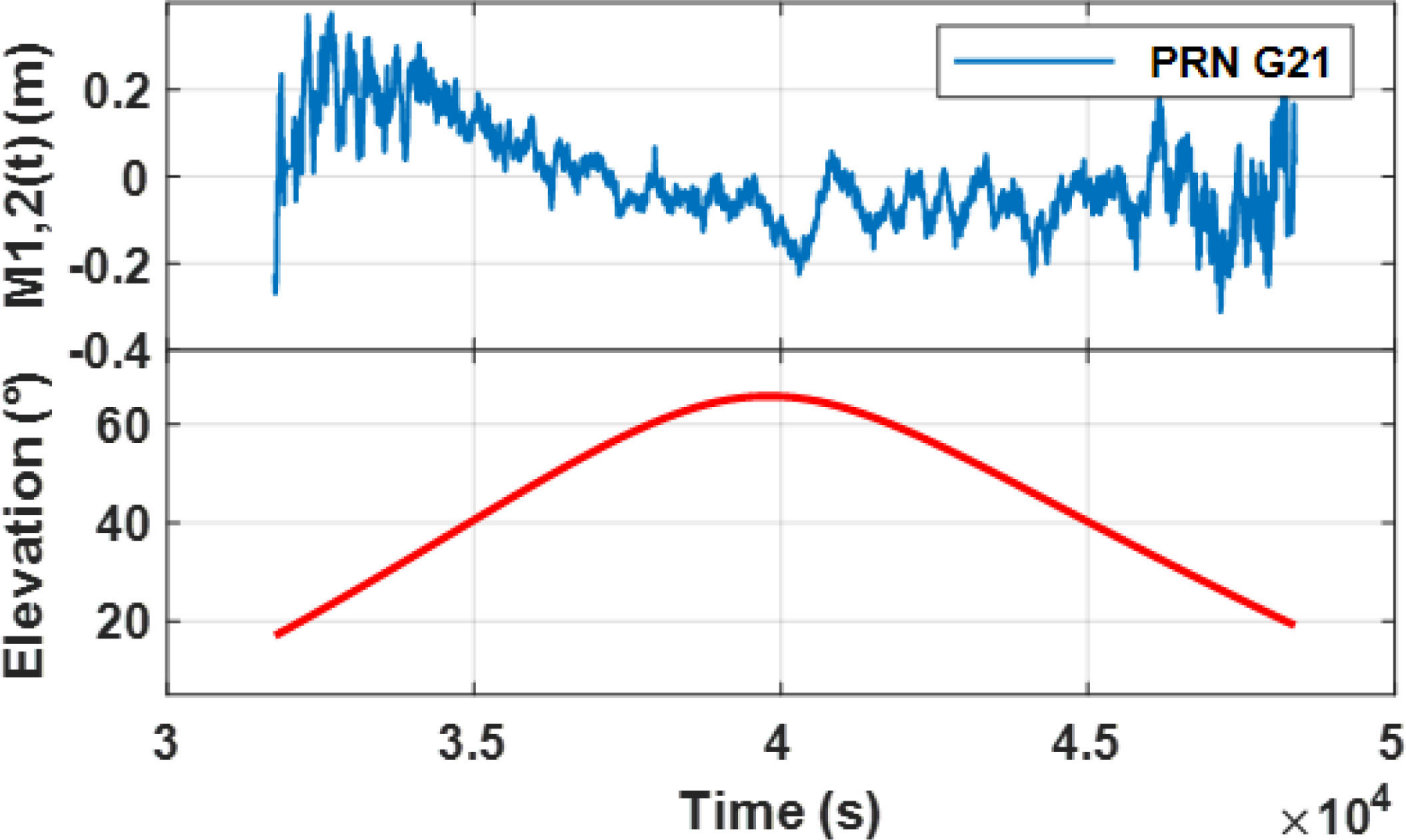
Sequence Combination with pseudorange and dual-frequency carrier phase observables for GPS PRN21.

**Figure 3:**
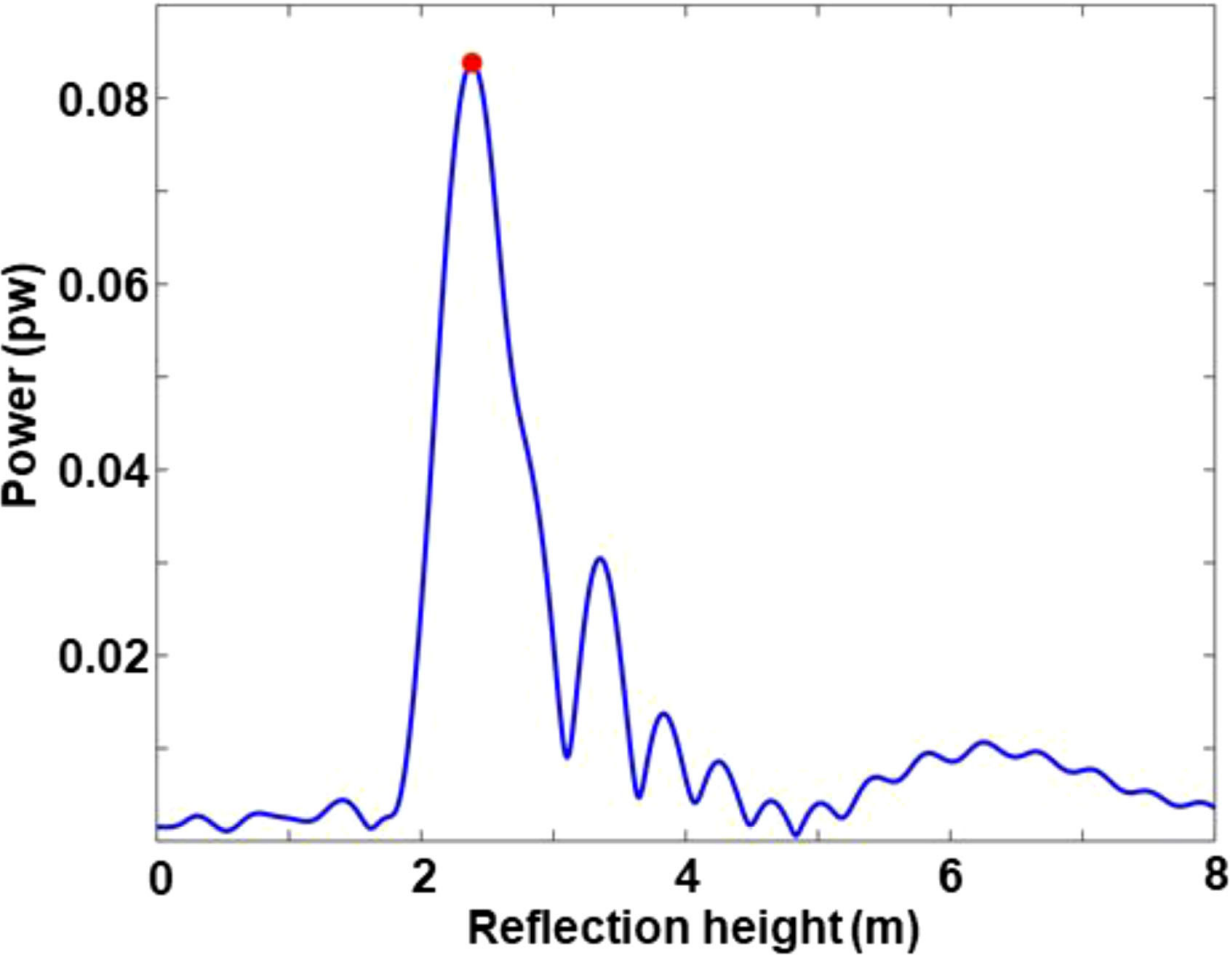
LSP analysis results for GPS PRN21.

Upon combining Equations 2 through 5 with Equation 9, it becomes apparent that the combined observation exhibits periodic fluctuations with the sine of the satellite elevation angle serving as the independent variable, showcasing non-uniform characteristics within the time domain. As a result, the conventional Fast Fourier Transform (FFT) technique proves inadequate for spectral analysis in this study. In contrast, the Lomb-Scargle Periodogram (LSP) method is adept at conducting frequency analysis on irregularly sampled signals, adeptly extracting subtle periodic signals while mitigating spurious signals to a certain extent (Wang et al., 2018). Moreover, this method can determine the false alarm probability and significance of each signal component. Therefore, this study leverages the LSP spectral analysis approach to identify the dominant frequency f within the combined observation sequence. Subsequently, this frequency data is translated into the vertical reflection height h utilizing Equation 11 and then integrated into Equation 1 to ascertain the wheat height $H_{\text{veg}}$. **Figures 2** and **3** respectively show the sequence combination and LSP analysis results of the observable combination for GPS PRN21.

### 2.3 Dual-system multi-satellite wheat height fusion method

Due to the variability in reflection point tracks and retrieval performances among GNSS satellites, wheat height retrieval reliant solely on a single system or type of combination often exhibit diminished reliability and inadequate stability. To tackle this challenge, the current study adopted the principal frequency power weighting to amalgamate retrievals from multiple satellites within a single system and employed the residual reciprocal weighting to blend results from GPS and BDS systems.

The power of the principal frequency plays a pivotal role in determining the reliability of the retrieval process. The presence of measurement noise can notably diminish this power, leading to a deviation of the peak frequency from its noise-free counterpart and subsequently compromising the accuracy of the retrieval. Consequently, prioritizing retrievals characterized by higher principal frequency power is anticipated to bolster both the stability and accuracy of wheat height estimation. The specific calculation methodology is expounded below.

The wheat height retrievals for different signal combinations from nsatellites of a single system can be expressed as Equation 12:

(12) $$\begin{array}{c} H_{sys}=\sum_{i=1}^{n}\frac{\sqrt{P_{i}}h_{i}}{\sum_{i=1}^{n}\sqrt{P_{i}}} \end{array}$$

where $P_{i}$ represents the dominant frequency power value obtained from LSP analysis from the i-th satellite, and $h_{i}$ denotes the retrieved wheat height based on the i-th satellite. The fused retrieval result from different systems can be expressed as Equation 13:

(13) $$\begin{array}{c} H_{fusion}=\sum_{j=1}^{k}H_{sys-j}\times w_{j} \end{array}$$

where $w_{j}\;$ represents the weight assigned to the retrievals from different systems, and its expression is given by and its expression is given by Equation 14:

(14) $$\begin{array}{c} w_{j}=\frac{\sigma_{j}{-}^{1}}{\sum_{j=1}^{k}\sigma_{j}{-}^{1}} \end{array}$$

where $\sigma_{j}$ represents the daily root mean square error between the fusion result of each GNSS system and the true or theoretical value.

## 3 Data and processing

### 3.1 Experiment and data

**Figure 4:**
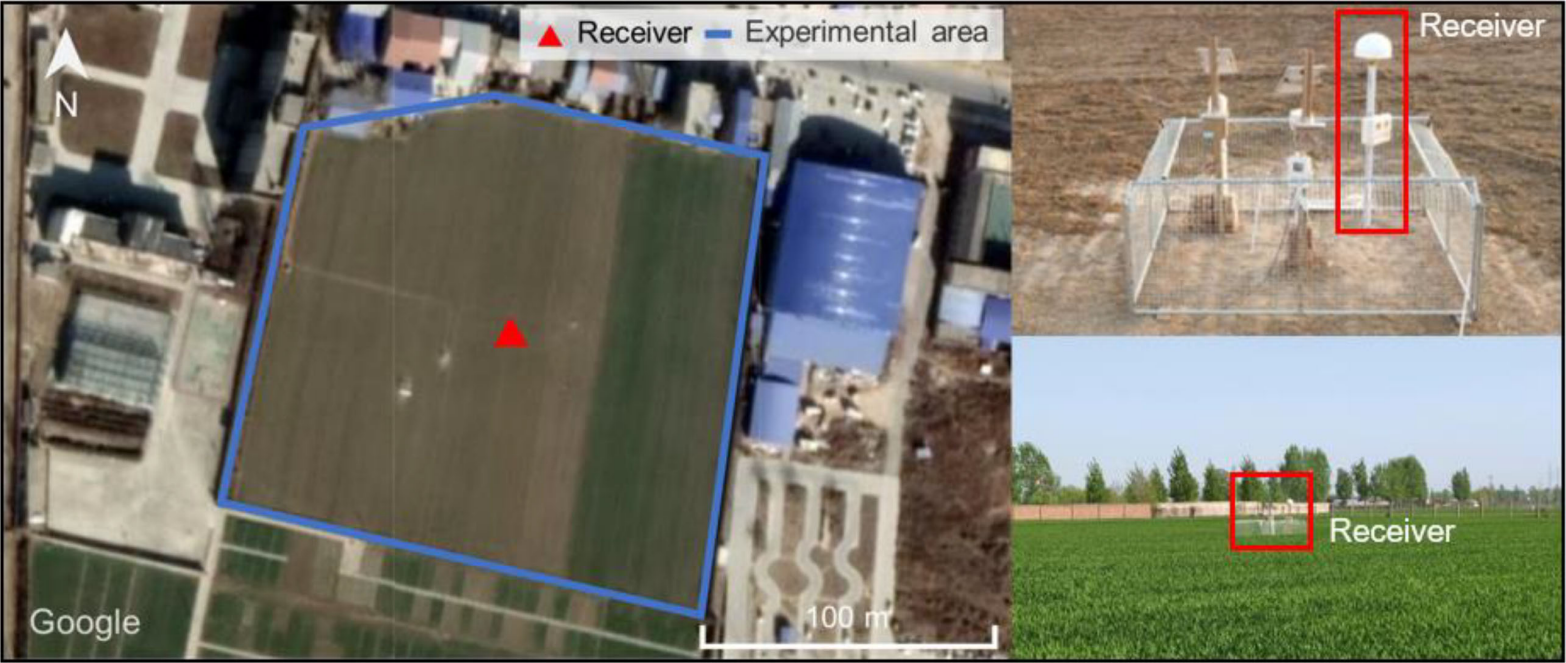
Surrounding environment and equipment at HNFQ.

The field experiment was conducted at the Fengqiu Agro-ecology Experimental Station (HNFQ) of the Chinese Academy of Sciences in Fengqiu County, Henan Province, China (35.019°N, 114.548°E). The site features open visibility, flat terrain, and negligible slope, which are favorable for stable GNSS multipath conditions (see **Figure 4**). Winter wheat was cultivated during the study period. A Sino M300 Pro GNSS receiver paired with an AT500 antenna continuously tracked multi-GNSS signals from January 1 to July 21, 2022 (DOY 1–172). A power outage caused data loss on DOY 157–167, and by DOY 156 the wheat had been harvested with no subsequent regrowth observed.

*In-situ* wheat height was measured at five-day intervals from the seedling stage (DOY 64) through maturity (DOY 133). At each measurement campaign, a metallic tape measure was used at four evenly distributed sampling locations within the field, and the arithmetic mean of these readings was taken as the daily effective height (**Table 1**). The initial measured height was 0.273 m, and the maximum height reached 0.7083 m on April 25 (DOY 115). For GNSS data acquisition, the receiver sampling rate was set to 15 Hz, and the observation types employed are summarized in **Table 2**. A low-elevation mask of 5° was applied during preprocessing to minimize multipath interference from ground clutter and nearby obstacles, and to exclude low-SNR observations that could adversely affect retrieval accuracy. This threshold was chosen based on empirical tests and aligns with many previous studies (Yu et al., 2018; Zhang et al., 2020; He et al., 2025).

**Table 1:** *In-situ* wheat height records.

**DOY**	64	69	74	79	84	89	95
**Wheat height (cm)**	27.3	28.2	32	35.94	42.17	42.83	52
**DOY**	100	105	110	115	120	124	133
**Wheat height (cm)**	54.33	56.5	69.98	70.83	70	69.38	70

**Table 2:** Types of observable used in the experiment.

Satellite system	Frequency band	Frequency (MHz)	Pseudorange	Carrier phase	SNR
GPS	L1	1575.42	C1C	L1C	S1C
	L2	1227.60	C2P	L2P	S2P
	L5	1176.45	C5I	L5I	S5I
BDS	B1-2	1561.098	C2I	L2I	S2I
	B3	1268.52	C6I	L6I	S6I
	B2b	1207.52	C7X	L7X	S7X

To mitigate blockage and ground-clutter effects from field margins and nearby features, low-elevation observations (elevation< 5°) were excluded during preprocessing.

Additionally, because manual measurements were available only every five days, a continuous daily reference series was constructed using a Growing Degree Day (GDD)-parameterized Logistic growth model. The model was fitted to the available *in-situ* measurements to produce daily wheat height estimates across the growing season, which served as reference values for evaluating the GNSS-based retrievals. The GDD model is described in detail in reference (Mahbod et al., 2014). Although it is acknowledged that such parametric model is a simplification and may deviate from true ground conditions under meteorological stress, disease pressure, or atypical weather patterns, it provides a reasonable continuous benchmark for evaluating retrievals over the growing season.

### 3.2 Retrieval step

**Figure 5:**
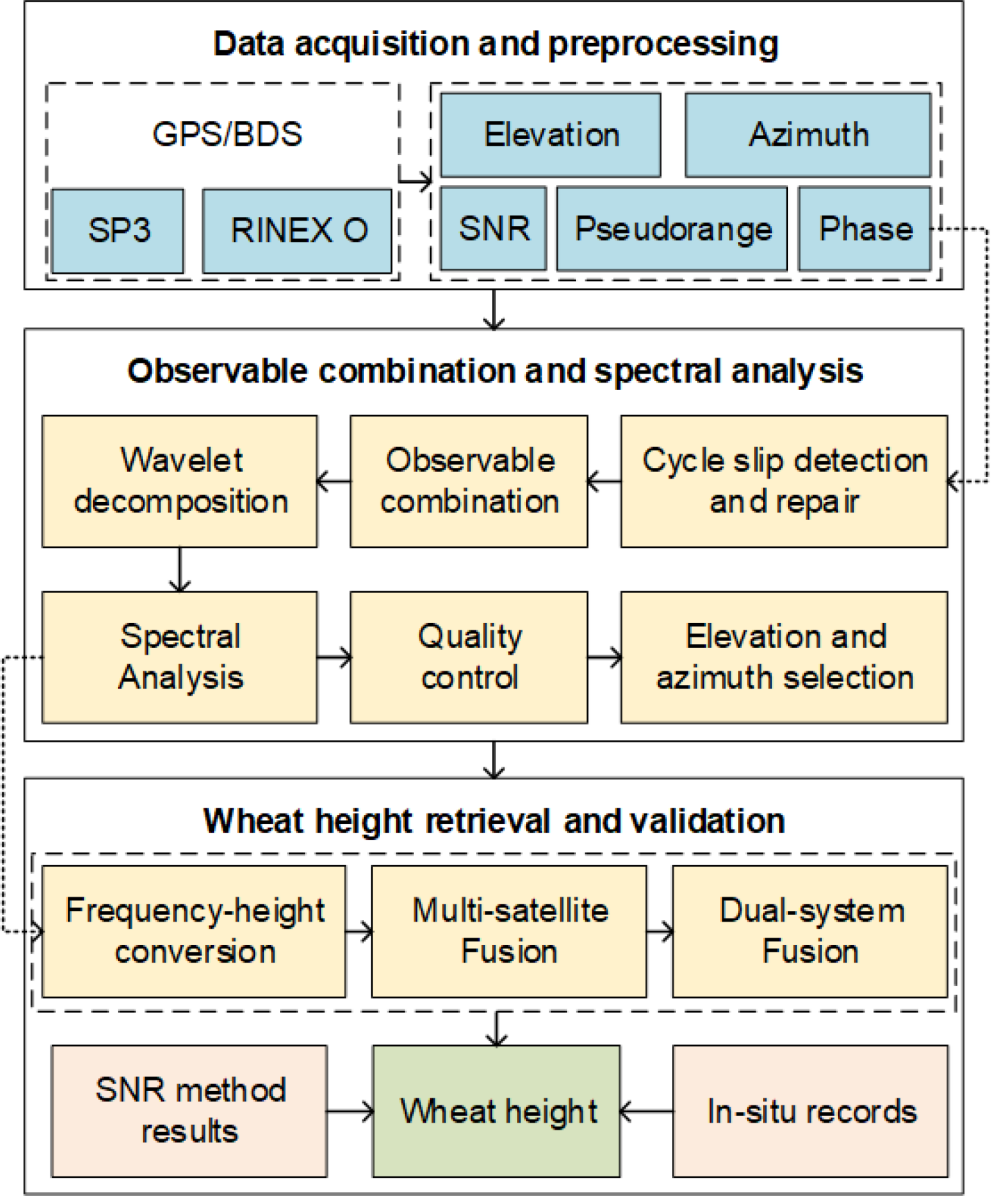
Flowchart of the retrieval algorithm process.

As illustrated in **Figure 5**, the workflow begins by ingesting RINEX observation files and precise ephemerides (SP3) from the experimental station. Pseudorange and carrier phase observables are extracted from these inputs, and satellite geometry (azimuth and elevation) is computed alongside the SNR for benchmarking purposes.

Subsequently, detecting and rectifying carrier-phase cycle slips, as well as screening outliers, are integral steps. The observables are then linearly combined following the combination scheme to amplify multipath sensitivity while dampening common-mode errors. To further improve signal quality, the combined time series is denoised and detrended using wavelet decomposition, employing a 5-level Daubechies 4 wavelet with soft thresholding. Considering the local environment, only arcs falling within geometry ranges conducive to coherent ground reflections (elevation 5–25°; azimuth 30–360°) are retained. The lower elevation bound mitigates atmospheric and obstruction effects, while the upper bound ensures sufficiently strong multipath oscillations.

Next, the combined sequences are spectrally analyzed using the Lomb–Scargle periodogram (LSP), which accommodates irregular sampling and highlights quasiperiodic interference signatures. The primary spectral frequency is pinpointed and translated into the vertical distance from the antenna phase center to the reflecting surface (wheat canopy). Through the geometric reflector-height model, this value is converted into per-arc canopy height estimates. Robustness is ensured by applying peak-power and false-alarm probability thresholds (set to 0.01 in this study), coupled with the elimination of residual outliers, culminating in a reliable daily compilation of wheat height retrievals.

To enhance stability and accuracy, we implement multi-source data fusion. Within each constellation, per-satellite estimates are weighted based on the spectral peak power. Across constellations, weights are determined following the method of reciprocal of residuals. The final dual-constellation, multi-satellite fused heights are produced by the weighted average.

Finally, we compare the height estimates obtained from single-constellation combinations and from the dual-constellation fusion against a conventional SNR-based approach processed in parallel. This comparative analysis validates the effectiveness of the combination-based retrieval and demonstrates the superiority of the multi-satellite, multi-constellation fusion for SNR-independent wheat height monitoring.

## 4 Results and discussion

### 4.1 Visual evaluation

The observable combination method was applied to retrieve wheat height using pseudorange and dual-frequency carrier phase combinations from both GPS and BDS at the experimental station. In order to better compare the performance of the retrievals obtained from the observable combinations from different systems and different signals, we designed six different combination schemes (**Table 3**). Since the pseudorange is a code-based measurement, it is inherently noisier than the carrier phase and is often the dominant source of error in the combination. Therefore, the differences among these schemes mainly lie in the selection of pseudorange observables.

**Table 3:** Combination schemes for GPS and BDS.

System	Pseudorange	Carrier phase
GPS	C1C	L1C/L2P
	C2P	L1C/L2P
	C5I	L5I/L2P
BDS	C2I	L2I/L6I
	C6I	L2I/L6I
	C7X	L6I/L7X

**Figure 6:**
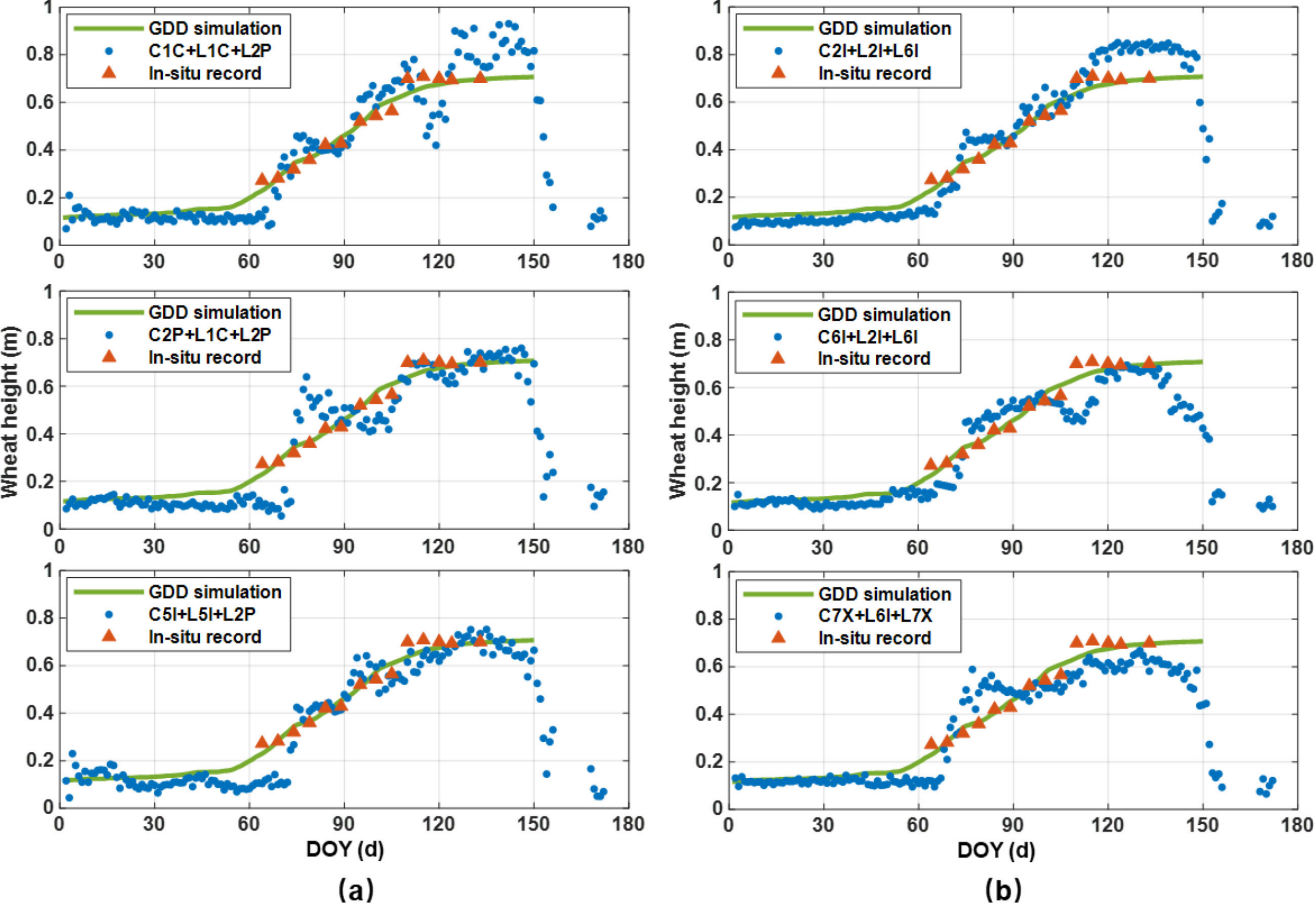
Wheat height retrievals from GPS **(a)** and BDS **(b)** signals.

The retrievals were compared against *in-situ* records and simulations derived from the GDD model. As depicted in **Figures 6a, b**, while discrepancies were evident among retrievals from different observable combinations, overall trends aligned well with wheat growth patterns. Fourteen *in-situ* measurements were gathered at five-day intervals from DOY 65 to 135. The retrieval patterns across all combinations concurred with the limited *in-situ* observations and the GDD model, effectively capturing key growth stages: winter wheat entered its stem elongation phase post-DOY 60, reaching a peak canopy height of approximately 0.7 m around DOY 120. This height remained stable until senescence commenced near DOY 156. Subsequent to harvest, a swift decline in measured height occurred during the post-harvest period (DOY 168-172), affirming the monitoring capability of multi-frequency GNSS-IR in tracking wheat height dynamics.

In addition, analysis of **Figures 6a** unveils notable accuracy discrepancies among GPS combinations. The C5I+L5I+L2P combination exhibited superior performance. Accuracy levels also fluctuated across growth stages. For instance, the GPS C1C+L1C+L2P and BDS C2I+L2I+L6I combinations displayed good agreement during jointing but tended to overestimate height during heading and maturation. It is proposed that this disparity might be attributed to the higher frequencies of the L1 and B1–2 signals (1575.42 MHz and 1561.098 MHz, respectively). Higher-frequency signals have shorter wavelengths and are more susceptible to scattering and attenuation in the upper canopy, reducing their penetration into lower layers. This results in a measured height closer to the canopy top, and when combined with the additional wavelength term in the model, may lead to overestimation (Zhang et al., 2021). In contrast, lower-frequency signals penetrate deeper, interacting with both canopy and underlying soil, thus providing more accurate height estimates during dense growth phases.

### 4.2 Quantitative evaluation

**Figure 7:**
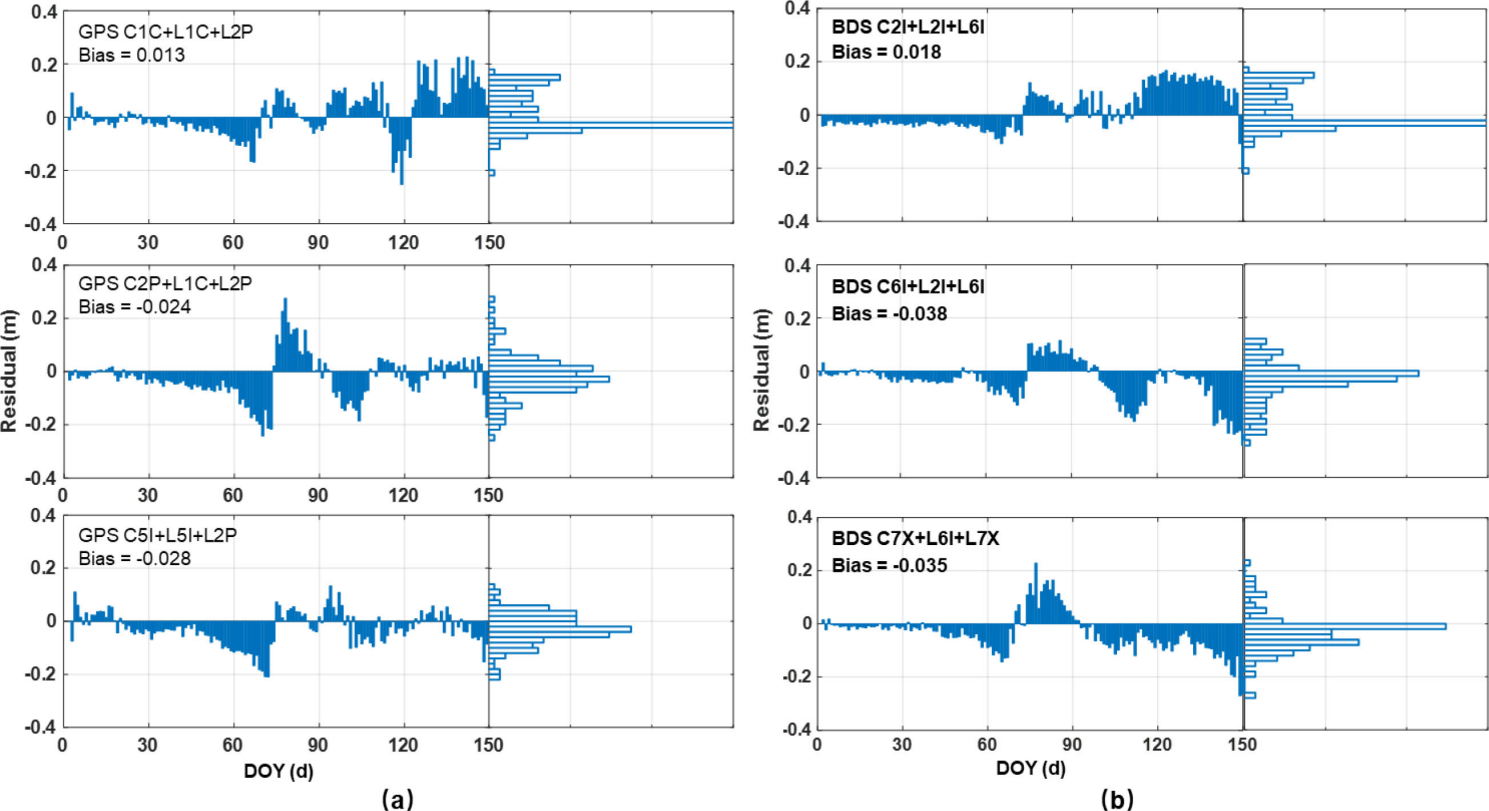
Residuals between wheat height retrievals and GDD simulations for GPS **(a)** and BDS **(b)**.

**Figures 7a, b** present the time-series deviations between the retrievals and GDD simulations for the GPS and BDS systems, respectively. Notably, the majority of residuals for both systems fall within the range of ±0.15 m. During the seedling stage (DOY 1–65), the retrieval residuals are minimal but gradually increase. In this period, the signal reflection surface is primarily a mixture of soil and wheat seedlings, while vegetation height retrieval is sensitive to height changes of at least one wavelength. Consequently, as the wheat grows, the residuals gradually expand with a clear negative bias becoming evident.

Transitioning into the jointing stage (DOY 66–110), the retrieval residuals exhibit more pronounced fluctuations, slightly surpassing those observed in the preceding growth stage. Rapid wheat growth leads to wider plant spacing, facilitating deeper signal penetration and introducing instability into the wheat height retrieval results. These dynamics account for the significant discrepancies between the inversion results and the reference values on specific days.

Beyond DOY 110, as the wheat progresses into the heading and maturity stages, distinctive patterns emerge. For the GPS system, the C1C+L1C+L2P combination retrieval residuals are relatively elevated, concentrated between 0.1–0.2 m. In contrast, other GPS combinations exhibit higher accuracy during this period, with the majority of errors falling within ±0.06 m. Within the BDS system, the inversion results of the C2I, L2I, and L6I combination slightly exceed the reference values, while other combinations show a notable negative bias compared to the reference during this phase.

An integrated analysis of **Figures 6** and **7** suggests that the BDS system demonstrates superior retrieval performance during the early growth and jointing stages of wheat. However, post-heading stage, its retrieval accuracy slightly lags behind that of the GPS system, underscoring the nuanced performance variations across growth stages and satellite systems.

**Figure 8:**
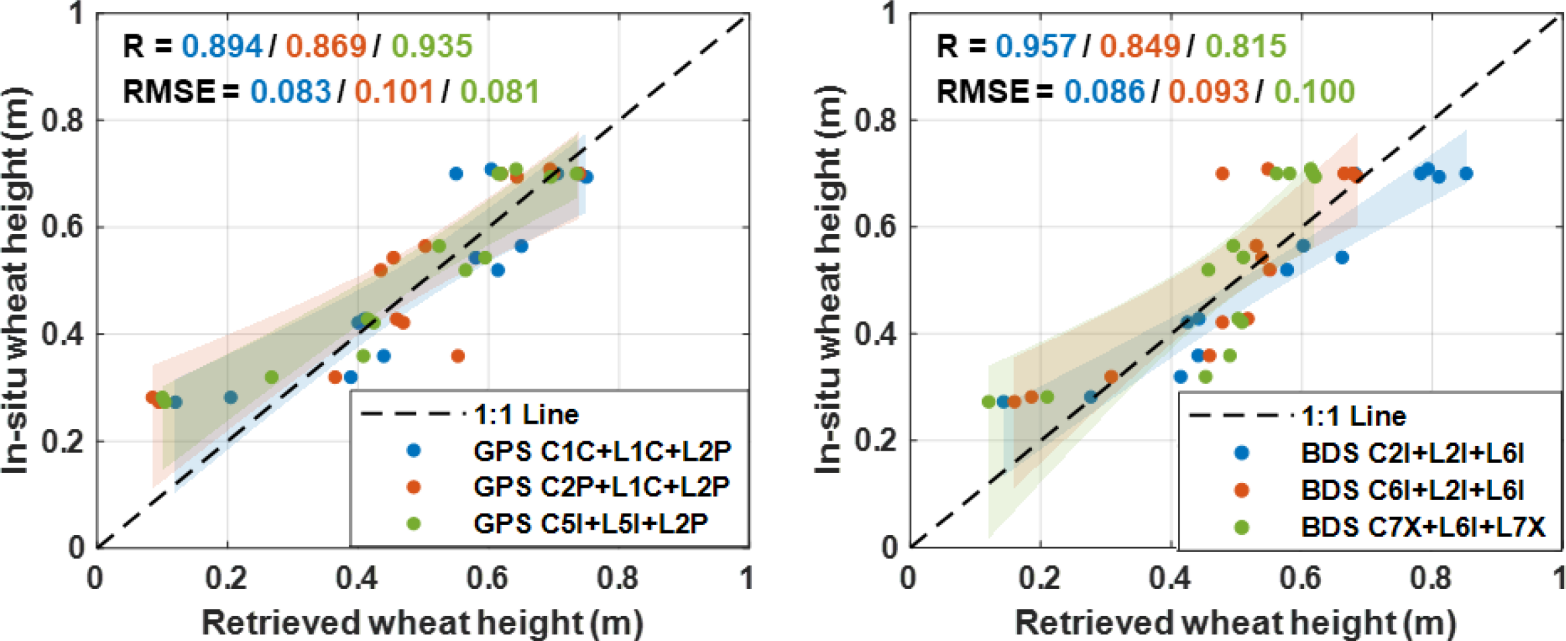
Scatter plots with confidence interval from wheat height retrievals and *in-situ* records for GPS and BDS.

**Figure 8** displays the scatter plots with confidence intervals from wheat height retrievals against *in-situ* records for GPS and BDS, respectively. Similar to the results shown in **Figures 6** and **7**, better agreements can be seen from GPS C5I+L5I+L2P and BDS C2I+L2I+L6I combinations. Specifically, the R and RMSE values are 0.935 and 0.081 for the former, and 0.957 and 0.086 for the latter, respectively. These two combinations show superior performance compared to the others. In addition, from the confidence intervals, it can be seen that the retrievals from all six combinations show improved consistency at greater wheat heights, with the GPS C1C+L1C+L2P and BDS C2I+L2I+L6I combinations performing better in this range.

### 4.3 Dual-system multi-satellite fusion results

To fully leverage the complementary advantages of multiple combinations across different satellites and systems, this study integrates multi-satellite retrievals from both GPS and BDS systems using the method proposed in section 2.3.

**Figure 9:**
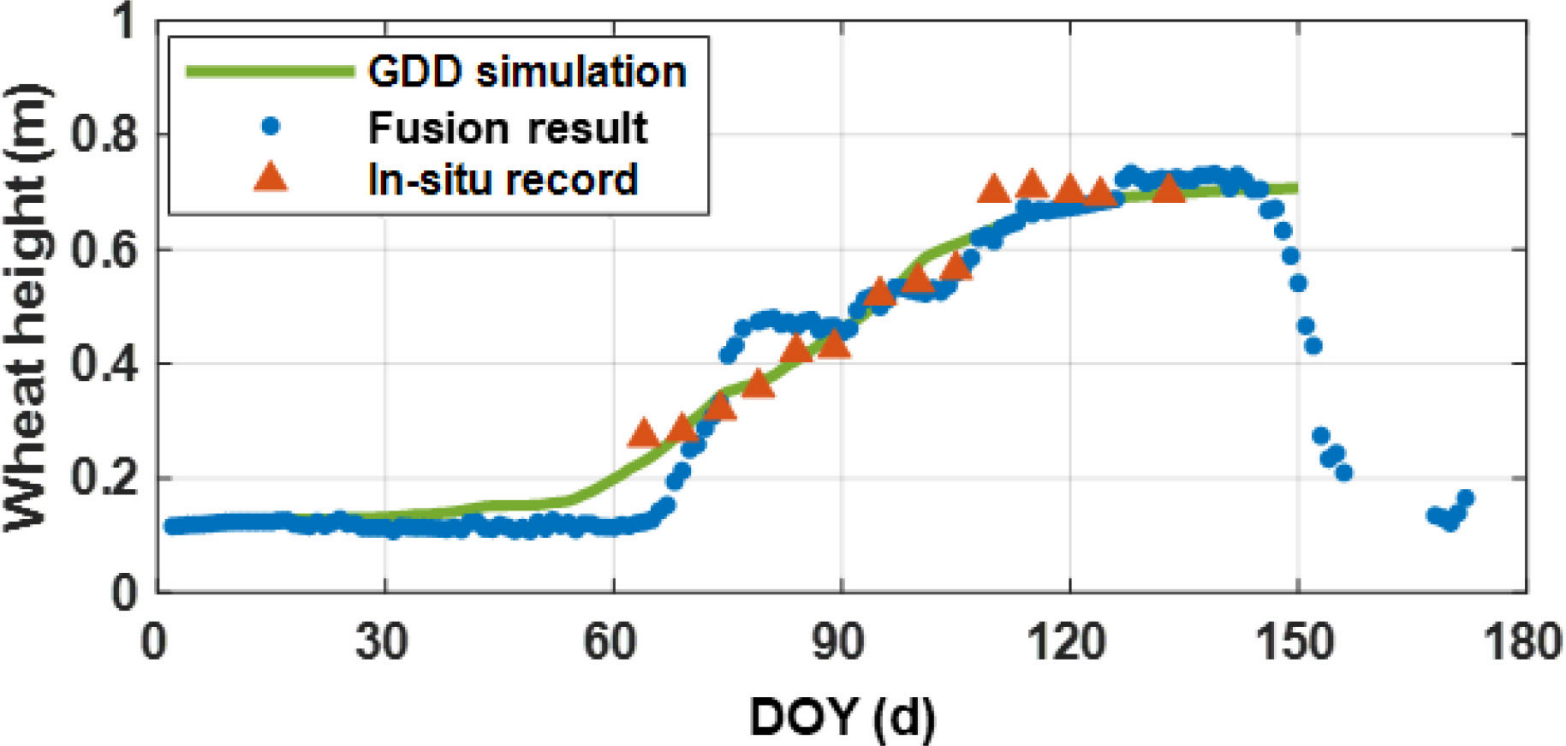
Dual-system multi-satellite wheat height fusion results.

**Figures 9** present the time series of fusion results, *in-situ* records and GDD simulations. As shown in the figure, the fused wheat height exhibits. The fused wheat height shows good consistency with both reference values. Compared to single-system results, the fusion retrievals demonstrate better agreement with the reference values. Notably, a high degree of consistency is observed with the GDD simulations during the periods of DOY 1–60 and 110–157, indicating that the data fusion method effectively utilizes the differences among individual systems and various combinations to enhance the accuracy of wheat height retrieval.

**Figure 10:**
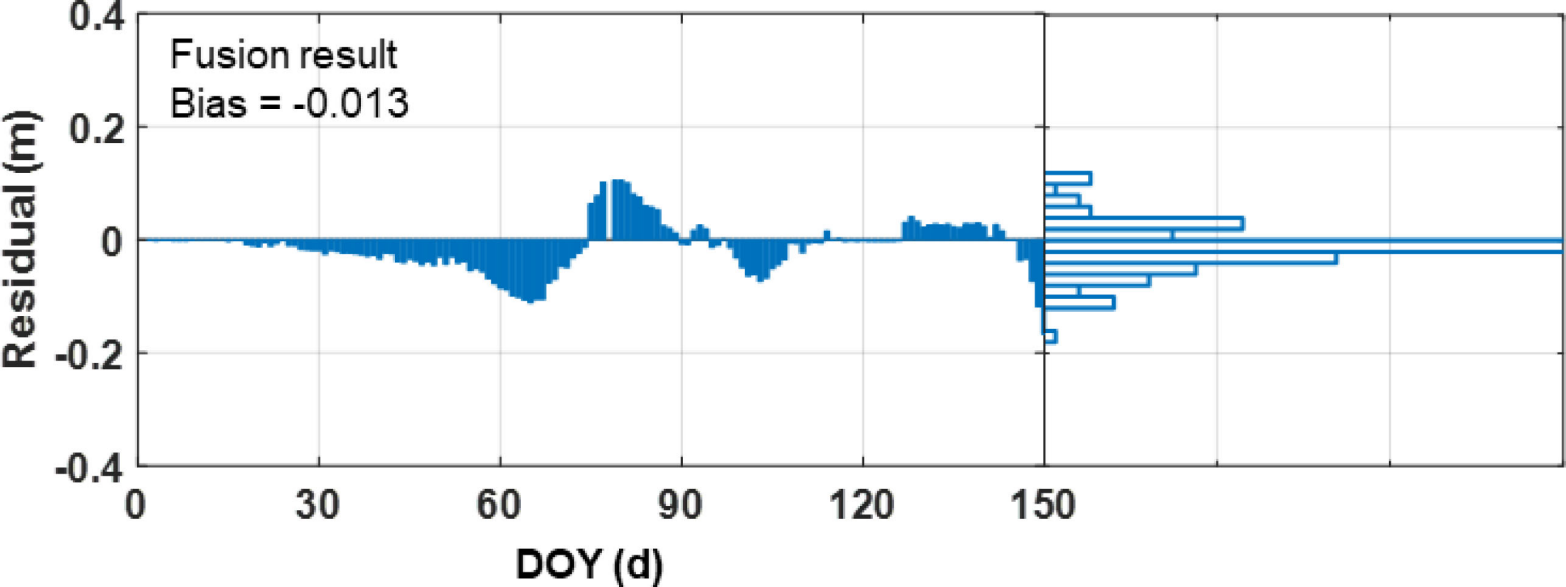
Residuals between fusion results and GDD simulations.

**Figure 11:**
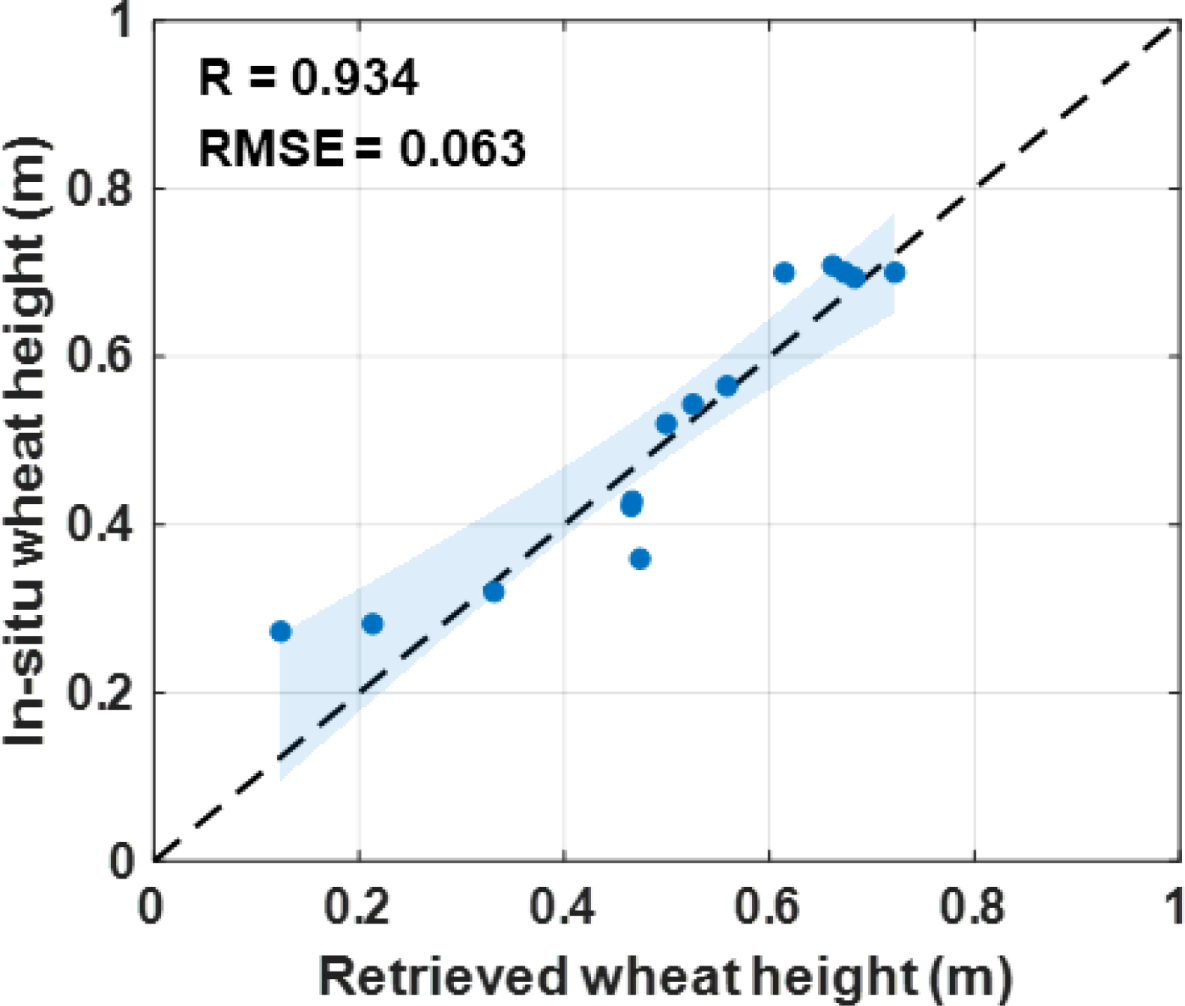
Scatter plots with confidence interval from wheat height fusion results and *in-situ* records.

Furthermore, the comparison of residuals with GDD simulations and scatter plots with *in-situ* records are displayed in **Figures 10** and **11**. Specifically, the fused wheat height exhibits a strong correlation with the *in-situ* records, with R and RMSE values of 0.934 and 0.063 m, respectively. Also, the fusion results show good agreement with the GDD simulations, with a low bias value of -0.013 m.

### 4.4 Comparison with SNR method results

To further validate the efficacy of the observable combination method, this study conducted a comparison with the SNR method, as outlined in **Table 4**.

**Table 4:** Result comparison between observable combination method and SNR method.

Method	Satellite system	Observable	R	RMSE (m)	MAE (m)
Observable combination	GPS	C1C L1C L2P	0.894	0.083	0.071
		C2P L2P L1C	0.869	0.101	0.080
		C5I L5I L2P	0.935	0.081	0.062
	BDS	C2I L2I L6I	0.957	0.086	0.071
		C6I L6I L2I	0.849	0.093	0.070
		C7X L7X L6I	0.815	0.100	0.095
Fusion	GPS+BDS	Fusion	0.934	0.063	0.047
SNR	GPS	S1C	0.938	0.096	0.075
		S2P	0.662	0.125	0.104
		S5I	0.833	0.091	0.067
	BDS	S2I	0.946	0.110	0.092
		S6I	0.768	0.109	0.089
		S7X	0.661	0.127	0.104

The results in the table demonstrate that, in contrast to the traditional SNR method, the observable combination method generally exhibits improved performance in wheat height retrieval, evidenced by smaller RMSE and MAE values. The heights retrieved through the observable combination method exhibit correlation coefficients exceeding 0.80 when compared to *in-situ* records, with RMSE ranging from 0.08 to 0.10 m and MAE from 0.06 to 0.095 m. Among these combinations, C5I, L5I, L2P stands out for its superior accuracy, achieving a correlation coefficient of 0.94, with RMSE and MAE values of 0.081 m and 0.062 m, respectively. In contrast, the SNR method yields wheat height retrievals with RMSE falling in the range of 0.09-0.13 m and MAE from 0.07-0.11 m. Notably, the S2P signal displays slightly diminished retrieval accuracy with a correlation coefficient of only 0.66.

Overall, the dual-system multi-satellite fusion results demonstrate optimal performance in wheat height retrieval, with a correlation coefficient of 0.93, and RMSE and MAE values of 0.063 m and 0.047 m, respectively. Compared to the best single-system combined observation method (C5I L5I L2P), this presents a 22.6% reduction in RMSE and a 23.7% reduction in MAE. Furthermore, in comparison to the best SNR method (SIC), it showcases a 34.6% decrease in RMSE and a 36.9% reduction in MAE. These findings underscore the efficacy and superiority of the observable combination method in enhancing wheat height retrieval accuracy.

## 5 Conclusion

Different from traditional navigation, positioning and timing (PNT) services, GNSS has been widely explored as an efficient remote sensing tool, with GNSS-R technology being used for surface environmental monitoring. In this study, the wheat height monitoring was successfully achieved for the first time based on ground-based GNSS-R with code pseudorange and dual-frequency carrier phase observables. *In-situ* experiments conducted at the HNFQ station, along with GDD simulations, were employed to validate the retrievals.

Six observable combinations from GPS and BDS were constructed and utilized for wheat height retrieval. The retrieved heights demonstrated correlation coefficients exceeding 0.80 when compared to *in-situ* records, with RMSE ranging from 0.08 to 0.10 m and MAE from 0.06 to 0.095 m. Among these combinations, GPS C5I+L5I+L2P and BDS C2I+L2I+L6I exhibited superior accuracy, with R and RMSE values of 0.935 and 0.081 for the former, and 0.957 and 0.086 for the latter, respectively. Across various stages of wheat growth, the retrieval results from different combinations effectively captured the variations in wheat growth.

To further enhance the accuracy and reliability of wheat height monitoring, this study implemented principal frequency power weighting to integrate retrievals from multiple satellites within a single system and utilized residual reciprocal weighting to blend results from GPS and BDS systems. Compared to the best single-system combined observation method, this approach yielded a 22.6% reduction in RMSE and a 23.7% reduction in MAE. Furthermore, through comparison with the SNR method, the effectiveness of the observable combination method was reaffirmed, demonstrating a 34.6% decrease in RMSE and a 36.9% reduction in MAE compared to the best SNR method (S1C).

In conclusion, the observable combination method, coupled with multi-system fusion strategies, offers significant advantages and promising applications in wheat height monitoring. It serves as an effective tool for real-time and accurate monitoring of wheat growth trends. However, it is important to acknowledge that the current validation is based on a specific field experiment under particular environmental conditions. The performance of the method under different scenarios (such as varying soil types, topography, meteorological stressors) remains to be thoroughly investigated. Future endeavors will therefore focus on validating the robustness and generalizability through multi-site and multi-season campaigns. Additionally, upcoming research will explore the performance of different combinations from GPS, BDS, Galileo, and GLONASS, aiming to construct a wheat height retrieval model based on full-system multi-frequency signal fusion of GNSS to enhance the accuracy and stability of retrievals.

## Data Availability

The original contributions presented in the study are included in the article/supplementary material. Further inquiries can be directed to the corresponding author.
